# Magnetic resonance imaging findings of non-mass enhancements in the breast and their association with malignancy

**DOI:** 10.1097/MD.0000000000047854

**Published:** 2026-02-20

**Authors:** Fadime Guven, Mehmet Eren Ozturk

**Affiliations:** a Faculty of Medicine, Department of Radiology, Ataturk University, Erzurum, Turkey; b Department of Radiology, Erzurum Regional Training and Research Hospital, Erzurum, Turkey.

**Keywords:** breast magnetic resonance imaging, non-mass enhancement lesions, time-intensity curves

## Abstract

Our aim is to investigate the relationship between the MRI (Magnetic Resonance Imaging) features of non-mass enhancement (NME) lesions and malignancy. MRI findings of 200 and 2 NME lesions were retrospectively evaluated. Age, family history of breast cancer, lesion pathology results, and follow-up durations of the patients were obtained from medical records. Based on follow-up findings and pathology results, the lesions were classified into 3 groups as benign, high-risk, and malignant. The association between these groups and MRI features was analyzed statistically. Segmental (48.4%) and diffuse (85.7%) distribution patterns, as well as clumped (51.5%) and clustered ring (100%) enhancement patterns of NME lesions, were found to be associated with malignant lesions. According to the time-intensity curve, a Type 3 curve was associated with malignant lesions in 84.6% of cases. When evaluating the number of dynamic curves, lesions with multiple curves were considered malignant in 29.2% of cases. Regarding apparent diffusion coefficient (ADC) values, malignant and high-risk lesions had significantly lower ADC values compared to benign lesions. In this study, segmental and diffuse distribution patterns, clumped and clustered ring enhancement, Type 3 curve, and low ADC values were found to be associated with malignant lesions.

## 1. Introduction

Among the primary indications for breast magnetic resonance imaging (MRI) are cancer staging, screening for breast cancer in women at high-risk, and evaluation of response to neoadjuvant chemotherapy.^[1,2]^ In breast MRI, lesions are categorized as focus, non-mass enhancement (NME), or mass. Unlike masses or foci, which have a defined 3-dimensional structure, NMEs are characterized as nonspecific areas of enhancement.^[2]^ The most recent edition (5th) of the Breast Imaging Reporting and Data System® (BIRADS®) describes the morphological features of NME in terms of distribution and internal enhancement patterns. Internal enhancement patterns include homogeneous, heterogeneous, clumped, and clustered ring enhancement, while distribution patterns are classified as focal, linear, segmental, regional, multiple regions, and diffuse. However, BIRADS does not yet provide a definitive categorization system for NME patterns.^[3]^ In a study investigating the relationship between the enhancement and distribution patterns of NME lesions and malignancy, linear, segmental, heterogeneous, clumped, and clustered ring patterns were reported to be associated with a higher likelihood of malignancy.^[2]^ In their study on NME lesions, Liu et al demonstrated a significant association between malignancy and segmental distribution, clustered ring enhancement, low apparent diffusion coefficient (ADC) values, and Type 3 (washout) pattern on time-intensity curves (TIC).^[4]^

The aim of this study is to investigate the association between NME lesions observed on breast MRI and malignancy, based on their distribution and internal enhancement patterns, diffusion characteristics including ADC values, and TIC types. It is aimed to contribute to the correct guidance of the radiological approach by providing data for the standardization of NME lesion findings that show an increased risk of malignancy. Another feature investigated in our study was the association between NME lesions exhibiting multiple TIC patterns and malignancy, and to our knowledge, no previous studies have examined this parameter.

## 2. Materials and methods

### 2.1. Patients

Ethics committee approval for this retrospective study was obtained from Ataturk University Faculty of Medicine Ethics Committee (Date: September 27, 2024, Decision no: 06/69). In our study, breast MRI images with dynamic contrast enhancement of 1834 patients performed between 2016 and 2024 were reviewed. The inclusion criteria for patients were defined as women over the age of 18 with dynamic contrast enhancement breast MRI images and NME lesions observed in either breast. It was determined that there were 224 patients who met these criteria.

Among the 224 patients who met the exclusion criteria, the following were excluded from the study: 15 patients who had received neoadjuvant chemotherapy, radiotherapy, or a history of surgery in the same breast, as this could alter the appearance of the lesion; 5 patients with insufficient image quality for evaluation; and 3 patients with malignancies in other organs. Thus, 202 NME lesions in 201 patients were evaluated in the study. Patient age, family history of breast cancer, lesion pathology results, and follow-up duration were obtained from medical records. The patient ages ranged from 20 to 73 years.

## 2.2. MRI technique

Breast MRIs were performed using a 3-T system (Siemens, Erlangen, Germany) with a dedicated breast coil in the prone position. A standard imaging protocol was applied, including precontrast sagittal fat-saturated turbo spin-echo T2-weighted imaging, coronal short tau inversion recovery, transverse turbo spin-echo T1-weighted imaging, transverse diffusion-weighted imaging using single-shot echo-planar imaging, and transverse dynamic pre and postcontrast fat-saturated fast low-angle shot 3-dimensional sequences. Gadolinium-based contrast agent was administered intravenously into the cubital vein via an automated injector at a dose of 0.2 mmol/kg, followed by a 20 mL saline flush, at an injection rate of 2 mL/s. Dynamic imaging commenced after a fixed delay of 30 seconds postcontrast injection, with each sequence lasting approximately 60 seconds. A total of 5 dynamic series were acquired. diffusion-weighted imaging was performed prior to the dynamic series using the following parameters: time of repetition 4000 ms, time of echo 60 ms, slice thickness 4.0 mm, field of view 380 mm, and diffusion-sensitizing gradients with b-values of 0, 400, and 800 seconds/mm^2^.

## 2.3. Image analyses

**Figure 1.**
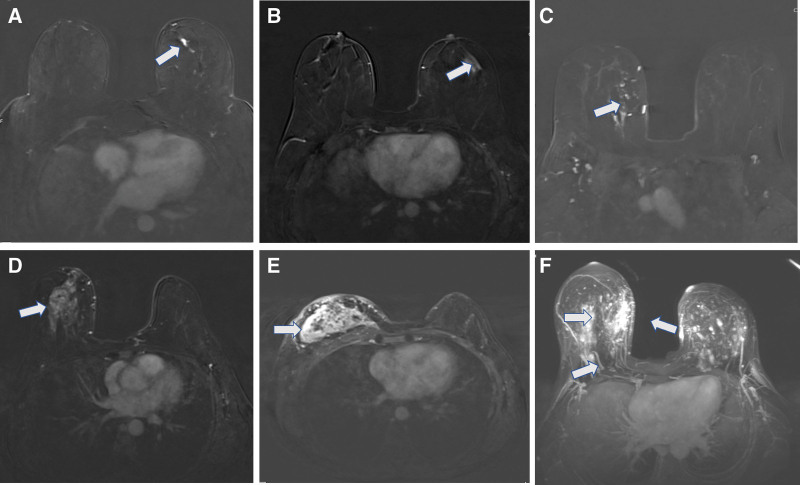
Distribution–contrast enhancement patterns; (A) linear homogeneous, (B) focal-heterogeneous, (C) regional-clumped, (D) segmental-heterogeneous, (E) diffuse-clustered ring, (F) multiple regions-heterogeneous.

**Figure 2.**
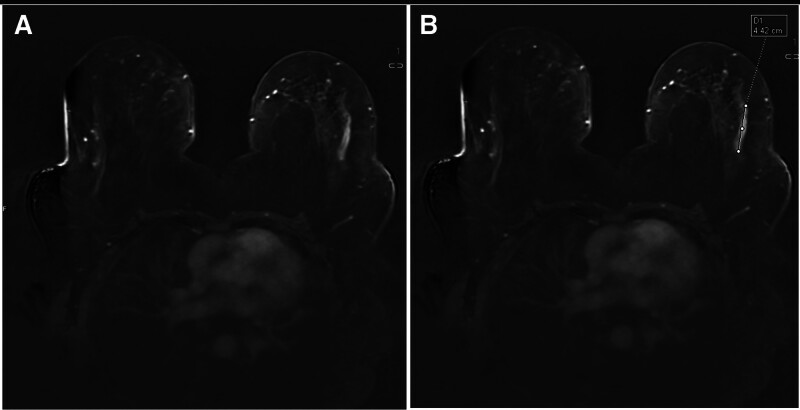
Measurement of linear pattern: (A) linear contrast enhancement pattern, (B) measurement of linear length.

**Figure 3.**
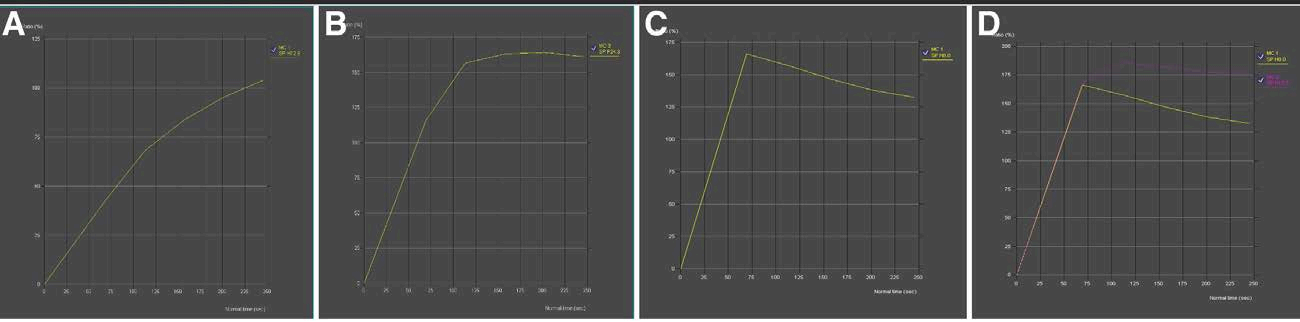
Time-intensity curves: (A) type 1 (persistent), (B) type 2 (plateau), (C) type 3 (washout), (D) multiple curves (type 2 and type 3).

Images were transferred from the PACS (Picture Archiving and Communication Systems) to a workstation (Syngovia, Siemens Healthineers, Germany) where analyses were performed. Image evaluations were conducted by 2 experienced radiologists (with 5 and 17 years of experience) in consensus, blinded to other imaging modalities, pathology results, and follow-up durations. The assessment of enhancement and distribution patterns was performed on axial contrast-enhanced sequences and 3D images, where the distinction between background parenchymal enhancement and NME lesions was most pronounced (Fig. 1). Distribution and enhancement patterns were defined according to the BIRADS 5th edition. In patients with linear enhancement patterns, the linear length of the NME lesion was measured in millimeters (Fig. 2). TIC measurements were performed on subtraction contrast-enhanced images, with measurements taken from at least 3 different regions within each lesion. The regions of interest were circular in shape and measured between 2 and 5 mm^2^ in area. TICs were classified as persistent (Type 1), plateau (Type 2), and washout (Type 3). The plateau pattern was defined as a delayed signal intensity change of equal to or <10%, while the washout pattern was defined as a delayed signal intensity decrease of more than 10%.^[1,5]^ Lesions exhibiting more than 1 TIC pattern were considered to have multiple curves (Fig. 3). In patients with multiple dynamic curves, the dominant curve was determined as the one with the highest enhancement pattern.

**Figure 4.**
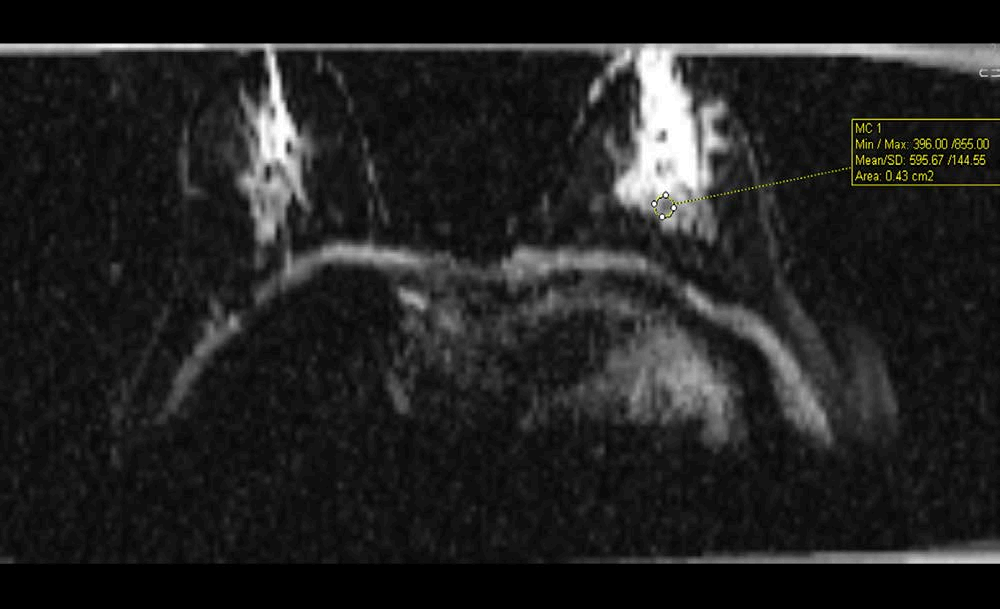
Measurement of ADC mean value. ADC = apparent diffusion coefficient.

ADC values were measured visually on ADC maps obtained from diffusion-weighted images by selecting 3 regions with the lowest ADC values. Among these 3 measurements, the lowest value was included in the study as the mean ADC value. The ADC regions of interest were circular with an area of 2 to 5 mm^2^ (Fig. 4).

## 2.4. Pathological and clinical evaluation of lesions

Lesions were categorized into 3 groups: benign, high-risk, and malignant, based on pathology and radiological follow-up findings. The malignant and high-risk groups included patients who underwent core needle biopsy, excision, lumpectomy, or mastectomy of the NME lesion and had a pathology report available in the system. The benign group included lesions with pathology reports available in the system confirming benign diagnosis (n = 68), lesions that remained stable during at least 24 months of follow-up (n = 101), or lesions that showed regression after 6 months or more of follow-up (n = 18). Malignant lesions included invasive carcinoma, invasive lobular carcinoma, and ductal carcinoma in situ. The high-risk group included atypical ductal hyperplasia, radial scar/complex sclerosing lesion, papillary lesion, and lobular carcinoma in situ. Benign lesions; fibrosis, fibrocystic changes, benign breast tissue, nonspecific inflammation, granulomatous mastitis were identified.

## 3. Statistical method

Descriptive statistics (count, percentage, mean, standard deviation, median, minimum, and maximum) were reported. When testing the relationship between categorical variables, Pearson Chi-Square test was applied when the expected cell count assumption (expected value >5) was met; otherwise, Fisher exact test was applied. Normality was assessed with the Shapiro–Wilk test and homogeneity of variances with the Levene test. When normality was not met, 2 independent groups were compared using the Mann–Whitney *U* test. For the comparison of 3 or more independent groups, one-way ANOVA was applied when the data were normally distributed; otherwise, the Kruskal–Wallis test was used. post hoc Bonferroni-adjusted tests were performed to identify the groups responsible for significant differences. Multinomial logistic regression was employed to model the categorical dependent variable with the independent variables. The analyses were performed using IBM SPSS version 27.

## 4. Results

The distribution of demographic and clinical variables according to pathology results was presented, and the relationships between them were analyzed using Pearson Chi-Square test and Fisher exact test. As a result of the analysis, statistically significant associations were found between pathology status and distribution pattern, enhancement pattern, dominant kinetic curve type and kinetic curve category (*P* <.001) (Table 1).

**Table 1:** Distribution of demographic and clinical measurements and their relationships according to pathology results.

		Benign			Intermediate high-risk			Malignant			*P*	Effect size
		n	%	%C	n	%	%C	n	%	%C		
Family history	No	125	86.8	84.5	5	3.5	71.4	14	9.7	82.4	.459	–
	Yes	23	82.1	15.5	2	7.1	28.6	3	10.7	17.6	–	–
Distribution pattern	Linear	77	96.3	46.1	0	0.0	0,.0	3	3.8	11.5	<.001*	0,677
	Focal	60	90.9	35.9	4	6.1	44.4	2	3.0	7.7	–	–
	Segmental	13	41.9	7.8	3	9.7	33.3	15	48.4	57.7	–	–
	Regional	15	88.2	9.0	2	11.8	22.2	0	0.0	0.0	–	–
	Multiple Regions	1	100.0	0.6	0	0.0	0.0	0	0.0	0.0	–	–
	Diffuse	1	14.3	0.6	0	0.0	0.0	6	85.7	23.1	–	–
Contrast enhancement pattern	Homogeneous	80	100.0	47.9	0	0.0	0.0	0	0.0	0.0	<.001*	0.660
	Heterogeneous	74	86.0	44.3	6	7.0	66.7	6	7.0	23.1	–	–
	Clumped	13	39.4	7.8	3	9.1	33.3	17	51.5	65.4	–	–
	Clustered Ring	0	0.0	0.0	0	0.0	0.0	3	100.0	11.5	–	–
Dominant kinetic curve type	1	93	95.9	55.7	3	3.1	33.3	1	1.0	3.8	<.001*	00609
	2	73	79.3	43.7	5	5.4	55.6	14	15.2	53.8	–	–
	3	1	7.7	0.6	1	7.7	11.1	11	84.6	42.3	–	–
Dynamic Curve Category	Single	136	88.3	81.4	6	3,.9	66.7	12	7.8	46.2	<.001*	0.281
	Multiple	31	64.6	18.6	3	6.3	33.3	14	29.2	53.8	–	–

%: row percentage, %C: column percentage for pathology results.

* *P* <.05.

** Pearson Chi-square test.

According to the distribution pattern, lesions with segmental and diffuse distributions showed higher rates of malignancy. Among lesions with a segmental distribution, 48.4% were associated with malignant pathology, while this rate was 85.7% for those with a diffuse distribution. In contrast, benign pathologies predominated in linear (96.3%), focal (90.9%), and regional (88.2%) distribution patterns (Table 1).

According to the enhancement pattern, all lesions with homogeneous enhancement were benign, while the malignancy rate was higher in lesions with heterogeneous and clumped enhancement patterns. Among lesions with a clustered ring enhancement pattern, all 3 were found to be malignant (Table 1).

According to the TIC analysis, 95.9 % of lesions with a Type 1 curve were classified as benign, whereas 84.6 % of lesions with a Type 3 curve were classified as malignant. Lesions exhibiting a Type 2 curve showed higher rates of intermediate and malignant pathology (Table 1).

According to the dynamic curve pattern, lesions exhibiting only a single curve had a higher benign rate (88.3%), whereas 29.2% of lesions with multiple curve types were classified as malignant (Table 1).

No statistically significant relationship was found between pathology results and family history (*P* >.05) (Table 1).

The distributions of age, ADC values, and linear length measurements according to pathology results are presented in the table, and comparisons were performed using ANOVA, Kruskal–Wallis, and Mann–Whitney *U* tests. The analyses revealed statistically significant differences in age and ADC values across pathology groups (*P* <.05) (Table 2).

**Table 2:** Distribution and comparison of age, ADC mean values, follow-up duration, and linear length measurements according to pathology results.

Measurements	Groups	Min–max	Mean ± SD (median)	*P*	Effect size
Age	Benign	20–65	40.10 ± 9.50 (40)	.002*	0.050
	Intermediate	23–54	38.78 ± 10.73 (40)	–	–
	Malignant	24–73	47.27 ± 11.1 (48.5)	–	–
ADC mean (mm^2^/s)	Benign	0.65–1.7	1.07 ± 0.19 (1.07)	<.001*	0.322
	Intermediate	0.6–1.1	0.834 ± 0.155 (0,8)	–	–
	Malignant	0.5–0.988	0.71 ± 0.126 (0,686)	–	–
Linear length	Benign	7–45	18.38 ± 9.65 (15)	.158	–
	Malignant	12–70	40.67 ± 29.01 (40)	–	–

ADC = apparent diffusion coefficient.

* *P* <.05.

† Kruskal–Wallis test.

According to the Bonferroni tests for age, a statistically significant difference was observed between the malignant and benign groups (*P* = .002); the malignant group was older than the benign group (Table 2).

According to the Bonferroni tests for ADC values, statistically significant differences were found between the benign group and both the malignant and intermediate groups (*P* <.001 and *P* = .005, respectively); ADC values in the benign group were higher than those in the malignant and intermediate groups (Table 2).

Multinomial logistic regression analysis was performed to model the effects of variables with statistically significant differences and impacts on pathology status. In the analysis, the benign group was taken as the reference category. The results indicated that among the variables of interest, ADC, age, and dominant kinetic curve had statistically significant effects on pathology status (*P* <.05). According to the goodness-of-fit test results, the obtained model was found to be statistically significant and suitable for use (*P* <.05). Considering that logistic regression models do not fully capture explanatory power, the Nagelkerke R^2^ value was examined, and the model was found to explain 66.4% of the variance. According to the classification table, the model correctly predicted 90.7% of the cases (Table 3).

**Table 3:** Multinomial logistic regression analysis for pathology status.

	Variables	β	SE	Wald	*P*	OR	95% CI	
							Lower bound	Upper bound
Intermediate	Constant	8.698	3.530	6.073	.014*			
	ADC	−0.008	0.003	9.890	.002*	0.992	0.987	0.997
	Age	−0.017	0.042	0.158	.691	0.984	0.907	1.067
	Dominant kinetic curve (1)	−3.415	2.034	2.819	.093	0.033	0.001	1.770
	Dominant kinetic curve (2)	−2.992	2.009	2.219	.136	0.050	0.001	2.572
	Dominant kinetic curve (3)	Ref.	–	–	–	–	–	–
Malignant	Constant	10.802	3.600	9.003	.003*	–	–	–
	ADC	−0.014	0.003	18.192	<.001*	0.986	0.980	0.993
	Age	0.100	0.043	5.375	.020*	1.105	1.016	1.202
	Dominant kinetic curve (1)	−7.520	2.324	10.468	.001*	0.001	5.694 × 10^−06^	0.052
	Dominant kinetic curve (2)	−5.067	2.049	6.115	.013*	0.006	0.000	0.350
	Dominant kinetic curve (3)	Ref.	–	–	–	–	–	–
	Model fit; X^2^ = 105,855 and *P* < .001*							
	Cox and Snell *R*^2^ = 0.453 and Nagelkerke *R*^2^ = 0.664							
	Correct classification rate = 0.907							

ADC = apparent diffusion coefficient, CI = confidence interval, OR = odds ratio, Ref = reference, SE = standard error.

* *P* <.05.

In the model for the intermediate group, the odds ratio for the ADC variable was calculated as 0.992 (*P* <.05). This indicates that when the ADC value increases by 1 unit, the likelihood of individuals being in the benign group is 1.008 (1/0.992) times higher than the likelihood of being in the intermediate group (Table 3).

In the model for the malignant group, the odds ratio for the ADC variable was calculated as 0.986 (*P* <.05). This indicates that for each 1-unit increase in ADC value, the likelihood of being in the benign group is 1.014 (1/0.986) times higher than the likelihood of being in the malignant group (Table 3).

The odds ratio for the age variable was found to be 1.105 (*P* <.05). This indicates that for each 1-year increase in age, the likelihood of being in the malignant group is 1.105 times higher than the likelihood of being in the benign group (Table 3).

The odds ratio for the dominant kinetic curve (1) was calculated as 0.001 (*P* <.05). When the dominant kinetic curve is Type 1 compared to when it is Type 3, the likelihood of individuals being in the benign group is 1000 (1/0.001) times higher than the likelihood of being in the malignant group (Table 3).

The odds ratio for the dominant kinetic curve (2) variable was calculated as 0.006 (*P* <.05). This indicates that when the dominant kinetic curve is Type 2, compared to Type 3, the likelihood of being in the benign group is 166.67 (1/ 0.006) times higher than the likelihood of being in the malignant group (Table 3).

Binary Logistic Regression analysis was applied to model the effects of pathology status on malignancy when the reference group for the measurements obtained in the study was intermediate, though a significant model was not obtained (Table 3).

## 5. Discussion

Some MRI findings observed in NME lesions are known to be associated with both malignant and benign entities. However, the categorization of NME lesions within BIRADS remains unclear, highlighting the need for further research. This study analyzes the breast MRI features of NME lesions to identify distinguishing characteristics among benign, high-risk, and malignant lesions.

In our study, morphological distribution assessment revealed that segmental (48.4%) and diffuse (85.7%) distribution patterns were associated with malignancy. The association of the segmental distribution pattern with malignancy aligns with findings from previous studies.^[2–4,6]^ A meta-analysis by Arian et al demonstrated that diffuse and multiple regions patterns had the highest odds ratios for malignancy.^[7]^ While this finding supports the high malignancy rate observed in the diffuse pattern in our study, the presence of only 1 multiple regions lesion limits the ability to draw definitive conclusions about the multiple regions pattern. In Aydin’s study, segmental and diffuse distribution patterns were also found to be associated with malignancy, consistent with our results.^[8]^ Similarly, Asada et al reported that all 3 NME lesions with diffuse distribution were malignant, corroborating our findings, though the small sample size may have influenced this outcome.^[3]^ Literature reviews confirm the association between segmental distribution and malignancy in multiple studies.^[6,9]^ This is thought to be due to malignant lesions spreading along ductal pathways.^[6]^ In our study, the diffuse distribution pattern was associated with malignancy in 85.7% of cases. However, some studies have not found a significant relationship between diffuse pattern and malignancy.^[4,6]^ We speculate that this discrepancy may be due to intense background parenchymal enhancement making differentiation difficult or the limited number of patients exhibiting the diffuse pattern.

Linear (96.3%), focal (90.9%), and regional (88.2%) distribution patterns were found to be associated with benign lesions. Consistent with our results, Aydin’s study showed that linear and focal patterns, and the study by Lunkiewicz et al demonstrated that linear, focal, and regional distribution patterns were the least associated with malignancy.^[8,10]^ However, the study by Cho et al demonstrated a significant association between the linear pattern and malignant lesions, which is inconsistent with our findings.^[9]^

Among the contrast enhancement patterns, homogeneous (100%) and heterogeneous (86.0%) patterns were significantly associated with benign lesions. This finding is consistent with studies in the literature.^[3,4]^ In the study by Yang et al, similar to our results, benign lesions were frequently observed with a homogeneous pattern, whereas, contrary to our findings, malignant lesions were more common in the heterogeneous pattern.^[11]^ The small number of heterogeneous cases in our study may have influenced this discrepancy. Regarding other enhancement patterns, clumped (51.5%) and clustered ring (100%) patterns were found to be associated with malignant lesions. The literature similarly associates clumped and clustered ring patterns with malignancy, aligning with our results.^[9,11]^ The fact that only 3 lesions exhibited the clustered ring pattern in our study may have contributed to the 100% association with malignancy. Therefore, larger studies with greater case numbers are clearly needed.

In our study, TIC analysis revealed that the Type 1 curve corresponded to benign lesions in 95.9% of cases, whereas the Type 3 curve was associated with malignant lesions in 84.6% of cases. Additionally, Type 2 curves demonstrated a higher malignancy rate compared to Type 1 curves. When examining the odds ratios using Type 3 as a reference, lesions exhibiting a Type 2 curve were 166 times more likely, and those with a Type 1 curve were 1000 times more likely to be benign. It is known that in breast lesions, the Type 3 curve is associated with malignancy, while the Type 1 curve is associated with benign lesions.^[1]^ The TIC results of studies on NME lesions are consistent with our findings.^[4,12]^ However, some studies have reported the highest positive predictive value for malignancy with Type 2 curves.^[8,11]^ This finding does not support the results of our study; however, in our study, the Type 2 pattern was found to be associated with malignancy at a higher rate compared to the Type 1 pattern. Future research should focus on a more detailed comparison between Type 2 and Type 3 patterns to better clarify their diagnostic significance.

In the TIC measurements, patients were evaluated based on whether the lesions exhibited single or multiple dynamic curves. Lesions with a single dynamic curve were associated with malignancy at a rate of 7.8%, whereas lesions with multiple dynamic curves showed a malignancy rate of 29.2% (*P* <.05). A review of the literature revealed no previous studies comparing the pathological characteristics of breast lesions based on whether their TIC curves are single or multiple. Our hypothesis is that the presence of multiple different dynamic curves in NME lesions may be related to the malignant nature of the lesion. Further studies are needed on this topic, involving both breast masses and NME lesions.

One of the findings of our study is the association between age and malignancy. A statistically significant difference was observed in the ages of patients in the benign and malignant groups. When examining the odds ratio for age, it was shown that with each 1-year increase in age, the likelihood of malignancy increased by 1.15 times, and the ages of patients in the malignant group were higher compared to the benign group (*P* <.05). It is well established that increasing age is a risk factor for breast cancer, supporting our findings.^[13]^ Additionally, some studies in the literature investigating NME lesions have also examined the relationship between age and malignancy, showing results similar to those of our study.^[14,15]^

It is known that family history is associated with malignancy in breast lesions.^[13]^ However, in our study, no association was found between family history and malignancy. This may be due to the lack of family history information in our retrospective review. Of the 30 patients with unavailable family history, 9 were diagnosed with malignant lesions, 2 with intermediate lesions, and 19 with benign lesions. The lack of family history for 9 of the 26 malignant lesions may have rendered the results insignificant.

In this study, a significant difference was found in ADC values (mm^2^/s) among benign, high-risk, and malignant lesions (*P* <.001). According to the odds ratios for ADC values, a 0.001 increase in ADC was associated with a 1.014-fold and 1.008-fold higher likelihood of being in the benign group compared to the malignant and intermediate groups, respectively. Our findings are consistent with the literature, where mean ADC values have been shown in various studies to be significant in differentiating malignant from benign lesions.^[16,17]^

No statistically significant difference was found between the length of the linear enhancement pattern of benign, high-risk, and malignant groups (*P* >.05). Similarly, Chen et al reported no significant difference in the positive predictive values for malignancy between linear lesions measuring less than or >1 cm.^[18]^

When considering the limitations of our study, the low number of lesions observed in certain patterns may have resulted in incomplete data. Therefore, studies with larger numbers of lesions are needed.

## 6. Conclusion

NME lesions are frequently observed among breast lesions but remain not clearly classified within the BIRADS system. The fact that the BIRADS classification of NME lesions has not yet been established makes breast MRI evaluation difficult and may lead to misdiagnosis. In our study, segmental and diffuse distribution patterns, clumped and clustered ring enhancement patterns, low mean ADC values, increased age, Type 3 kinetic curve, and the presence of multiple dynamic curves in NME lesions were found to be associated with malignancy. We believe that similar studies and future meta-analyses may contribute to the BIRADS categorization of NME lesions. Including NME lesion characteristics in the BIRADS classification will facilitate their radiological evaluation.

## Author contributions

**Conceptualization:** Fadime Guven.

**Formal analysis:** Mehmet Eren Ozturk.

**Investigation:** Mehmet Eren Ozturk.

**Methodology:** Fadime Guven.

**Resources:** Fadime Guven.

**Supervision:** Fadime Guven.

**Writing – original draft:** Mehmet Eren Ozturk.

**Writing – review & editing:** Mehmet Eren Ozturk.
